# Textural Features from Quantitative MRI Using T2 Relaxation Times Correlate with Histological Cartilage Degeneration: An Ex-Vivo Study

**DOI:** 10.1177/19476035261478556

**Published:** 2026-08-20

**Authors:** Markus Schreiner, Stefan Toegel, Veronika Janacova, Pavol Szomolanyi, Didier Laurent, Franziska Saxer, Matthias Schieker, Rahel Heule, Oliver Bieri, Esther Raithel, Christoph Fuchssteiner, Wolfgang J. Weninger, Reinhard Windhager, Siegfried Trattnig, Benedikt Hager, Vladimir Juras

**Affiliations:** 1 Department of Orthopedics and Trauma Surgery, 27271 Medical University of Vienna, Vienna, Austria; 2 Ludwig Boltzmann Institute for Arthritis and Rehabilitation, Vienna, Austria; 3 Department of Biomedical Imaging and Image-guided Therapy, High Field MR Centre, 27271 Medical University of Vienna, Vienna, Austria; 4 Christian Doppler Laboratory for MR Imaging Biomarkers (BIOMAK), Department of Biomedical Imaging and Image-guided Therapy, Medical University of Vienna, Vienna, Austria; 5 Department of Imaging Methods, Institute of Measurement Science, Slovak Academy of Sciences, Bratislava, Slovakia; 6 Department of Translational Medicine, 572186 Novartis Biomedical Research, Basel, Switzerland; 7 Medical Faculty, 27209 University of Basel, Basel, Switzerland; 8 Department of Health Sciences and Technology, ETH Zurich, Zurich, Switzerland; 9 Center for MR Research, University Children’s Hospital, Zurich, Switzerland; 10 Department of Biomedical Engineering, 27209 University of Basel, Allschwil, Switzerland; 11 Department of Radiology, Division of Radiological Physics, University Hospital Basel, Basel, Switzerland; 12 Research & Clinical Translation, Magnetic Resonance, 42406 Siemens Healthineers AG, Erlangen, Germany; 13 Division of Anatomy, Center for Anatomy and Cell Biology, 27271 Medical University of Vienna, Austria; 14 Austrian Cluster for Tissue Regeneration, Ludwig Boltzmann Institute for Experimental and Clinical Traumatology, Vienna, Austria

**Keywords:** cartilage, osteoarthritis, structural changes, texture analysis, GLCM, validation, PLM

## Abstract

**Objective:**

To assess the relationship between textural features derived from quantitative MRI (T_2_ mapping) and histological signs of cartilage degeneration.

**Design:**

Ten fresh non-frozen knees from body donors were measured at 7T and 3T scanners using 3D-Double Echo Steady State (3D-DESS) as well as 3D-Triple Echo Steady State (3D-TESS) sequence. The 3D-DESS images were automatically segmented and co-registered with T_2_ maps at both field strengths. Nine osteochondral plugs were harvested from standardized regions of the distal femur of each knee. The histological workup included Safranin O and picrosirius red staining. Mankin scoring was performed for each plug. Collagen orientation and parallelism were assessed and quantified based on polarized light microscopy. Each plug was segmented on the 3D model derived from 3D-DESS image and texture analysis was performed on the co-registered T_2_ map. Relationships between T_2_ relaxation time, textural features and the Mankin score, as well as collagen orientation and parallelism, were assessed using partial Spearman’s correlation coefficients and mixed models.

**Results:**

Mean T_2_ and six features (autocorrelation, contrast, correlation, entropy, sum entropy and inverse difference moment normalized) correlated with the MS; autocorrelation, contrast and entropy were also found to be correlated with collagen orientation. No features were significantly correlated with parallelism. Statistical models showed no significant effect of sequence or field strength in predicting Mankin score.

**Conclusions:**

The presented ex-vivo approach enables a semi-automated assessment of articular cartilage quality that correlates with histological measures of degeneration. Additional studies are warranted to further investigate the reproducibility and validity of the measurements.

## Introduction

Osteoarthritis (OA) remains a major global health challenge, with projected increase in cases by 74.9% for knee OA from 2020 to 2050.^1^ It is a degenerative multifactorial whole joint disease, affecting not only the knee joint but also the hip, ankle, wrist, shoulder, and other joints.^2^ Though the exact underlying pathological mechanisms are still not known, changes result in an imbalance between anabolic and catabolic processes in the joint tissues, which leads to cartilage deterioration, inflammation, bony overgrowths, biomechanical changes and pain.^3^ While imaging is not essential for clinical diagnosis, structural changes are typically evaluated using radiographs or magnetic resonance imaging (MRI), the latter being particularly suitable for evaluating soft tissues.^4^

When focusing on cartilage, early signs of OA might not be visible on routine MRI. At the tissue level, the metabolic imbalance in chondrocytes leads to cartilage matrix degradation, decline in proteoglycan synthesis, an increase in water content, and a disruption of its zonal organization.^5^ Healthy cartilage has a distinctive zonal structure on T_2_ maps, with higher T_2_ values in the superficial layer and lower T_2_ values near the bone-cartilage interface.^6^ With proteoglycan depletion, the increase in free water content is clearly visible on quantitative MRI T_2_ maps as areas with increased T_2_ values making T_2_ mapping a reliable non-invasive diagnostic technique for early matrix changes.^7^ The absolute T_2_ values depend not only on the cartilage integrity, but also on the imaging sequence used and field strength. The higher the field strength, the better is the signal-to-noise ratio, but the shorter are the T_2_ values.^8,9^ Thus, the ultra-high field improves the precision of voxel-wise T_2_ estimation, but images show less pronounced stratification of T_2_ in comparison to lower fields.^10^

Furthermore, the spatial distribution of the T_2_ values (i.e. the image texture) - can provide additional information about the cartilage state reflected by a spatial heterogeneity of T_2_ values as a result of the disruption of the zonal architecture in early cartilage degeneration. Gray-level co-occurrence matrix (GLCM) texture analysis has been explored in the context of detecting early OA changes.^11,12^ The GLCM quantifies the frequency of pixel intensity pairs occurring at a defined spatial relationship within an image. From this matrix, second-order statistical features can be derived, which characterize linear and quadratic dependencies between neighboring pixels and provide detailed information about tissue texture.^12^ While in vivo texture changes have been reported, direct validation against histology and collagen architecture remains limited. The Mankin score is a semi-quantitative histopathological grading system commonly used to classify OA severity based on cartilage structure, cellularity, proteoglycan depletion and tidemark integrity.^13^ To better assess the collagen network structure, polarized light microscopy (PLM) can be used to calculate quantitative parameters such as fiber orientation and fiber parallelism from histological samples.^14^

This study aimed to evaluate whether texture features derived from quantitative MRI T_2_ maps can serve as biomarkers of cartilage quality in early OA by comparing them against the histological reference standard (Mankin score) and complementary collagen organization measures. The analysis also compared texture-histology relationships between clinically standard 3T and ultra-high-field 7T MRI.

## Methods

This study was approved by the institutional ethics committee. Ten fresh, non-frozen knees from body donors (five females, mean age 80.4 ± 16.4 and five males, mean age 78.2 ± 9.3) were scanned in an investigational 7T MRI whole-body system (Siemens Healthineers, Forchheim, Germany) using a 28-channel knee array coil (QED, Quality Electrodynamics LLC, Cleveland, USA) and a 3T MRI whole body system MAGNETOM Prisma Fit (Siemens Healthineers, Forchheim, Germany) using an 8-channel knee array coil (Invivo, Gainesville, FL, USA). To preserve native tissue properties, the knees were not frozen at any time prior to imaging. Following death, the bodies were stored in a temperature-controlled environment at 4 °C. Magnetic resonance (MR) imaging was performed within 48 hours after death, first at 7T and immediately thereafter at 3T while keeping the knees in the airtight double plastic bags to ensure adequate hydration. The imaging protocol included a high-resolution 3D double-echo steady-state (3D-DESS) sequence for cartilage segmentation and quantitative sequences for T_2_ mapping, a 2D multi-slice multi-echo spin echo (MESE) on 3T and a 3D triple-echo steady-state (3D-TESS) sequence on both field strengths.^15,16^ MESE was not feasible at 7T because high specific absorption rate (SAR) constraints limited the whole-knee coverage by reducing the number of available slices and echoes.^17^ The sequence parameters are summarized in Table 1. The 3D DESS sequence is widely used in musculoskeletal (MSK) MRI for high-resolution imaging of cartilage, menisci, and joint structures. It combines two echoes—one with predominantly T_1_/T_2_* weighting and another with more T_2_ weighting—to provide excellent signal-to-noise ratio and tissue contrast. DESS enables thin, isotropic 3D acquisitions, allowing multi-planar reformatting without loss of detail.^18^ It is particularly useful for cartilage morphology assessment, joint fluid visualization, and bone–cartilage interface imaging.^19^ MESE sequence is the standard method for T_2_ mapping in cartilage and other MSK tissues. It acquires multiple spin-echo images at different echo times (TEs) to fit the T_2_ relaxation decay curve voxel by voxel.^20^ TESS sequence is a fast, steady-state alternative for T_2_ mapping. It acquires three echoes within a single steady-state acquisition, allowing simultaneous estimation of T_1_, T_2_, and proton density with shorter scan times and high signal-to-noise efficiency. TESS is less sensitive to B_1_ and B_0_ inhomogeneities and magic-angle effects than MESE, making it more robust for clinical and research applications.^15^

**Table 1.** MR Protocol and Sequence Parameters. 3D-DESS - 3D-Double Echo Steady State; 3D-TESS - 3D-Triple Echo Steady State, MESE - 2D Multi-Slice Multi-Echo Spin Echo

Field strength→	3T			7T	
↓Sequence parameters	3D-DESS	3D-TESS (T_2_ mapping)	MESE (T_2_ mapping)	3D-DESS	3D-TESS (T_2_ mapping)
Image plane	sagittal	sagittal	sagittal	sagittal	sagittal
Slice thickness	0.5 mm	3 mm	2.5 mm	0.5 mm	3 mm
Interslice gap	0.0 mm	0.0 mm	0.5 mm	0.0 mm	0.0 mm
Repetition time (TR)	13.95 ms	11.14 ms	2750 ms	8.68 ms	8.74 ms
Echo time (TE)	5.2 ms	5.53 ms^*^	[11.1, 22.2, 33.3, 44.4, 55.5, 66.6, 77.7, 88.8] ms	2.55 ms	4.43 ms^*^
Acquisition matrix	320 × 320	320 × 320	320 × 320	320 × 320	320 × 320
Field-of-view	165 × 165	160 × 160	160 × 160	160 × 160	160 × 160
Flip angle	30°	15°	180°	18°	15°
Bandwidth	275 Hz/px	445 Hz/px	200 Hz/px	345 Hz/px	501 Hz/px
Acquisition time	8:10 min	3:52 min	7:50 min	3:56 min	3:17 min

*With TESS, the three echoes (steady-state free precession configurations) were acquired in three consecutive TRs (one echo per TR) at identical echo times.^21^

**Figure 1.**
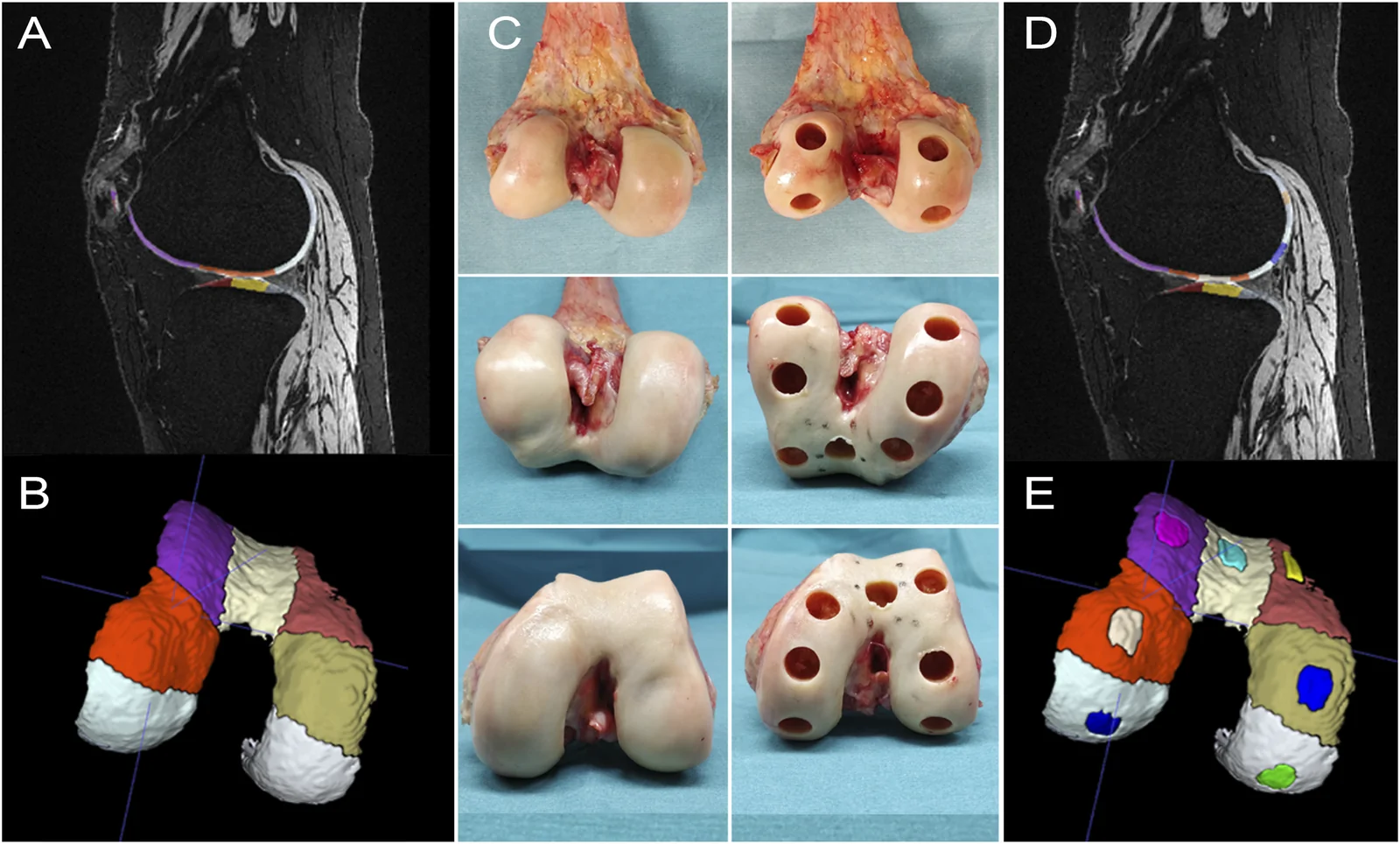
(A) and (B) results of an automated cartilage segmentation using the MRChondralHealth V3.1 research application, (C): a representative example of cartilage extraction for histological analysis (different views, before harvest on the left, after harvest on the right), (D) and (E): automated segmentation augmented by manual selection of plugs corresponding to the actually extracted plugs

MRChondralHealth V3.1 research application software (Siemens Healthineers, Forchheim, Germany; segmentation algorithms from Fripp et al.^22^ and Chandra et al.^23^) was used to segment the femoral cartilage and subdivide it into nine regions: medial anterior, medial central, medial posterior, trochlea lateral, trochlea medial, trochlea central, lateral anterior, lateral central, lateral posterior (Figure 1).^22,23^ Subsequently, nine cylindrical plugs were manually selected on the respective cartilage regions following the size and localization of the plug extraction as described in Figure 1. The patellar cartilage was not processed in the study because the kneecap was pulled distally due to the lack of muscle tension in the quadriceps tendon of the donor knees, which impaired automatic segmentation due to the atypical positioning. The tibial cartilage was not used because it was difficult to simultaneously remove the plugs from the femur and tibia from the corresponding locations without damaging them. To ensure the exact location of the plug on MRI images and plug extraction, the cylindrical plugs of size 10 millimeters were extracted from the center of the each of the nine cartilage regions following the anatomical landmarks according to Surowiec et al.^24^ Slice thickness allowed for up to nineteen slices matching the actual plug extraction, however, four slices on the edges (two on each side) were insufficient in size for the texture analysis and were omitted.

After the MR measurements, the knee joints were processed by an orthopaedic surgeon. After a midline incision, a medial parapatellar approach was used to gain exposure of the knee joint. Special attention was paid not to damage the cartilage during preparation. After exposing the distal femur and clearing it from any soft tissue, the femoral cartilage was subdivided according to the anatomical landmarks of the segmentation algorithm and an osteochondral plug was harvested from each region (Figure 1C) using an OATS (Arthrex, Naples, USA) instrument. The nine harvested plugs were added as separate regions of interest (ROIs) to the segmentation mask (Figure 1E) and then used for the T_2_ and GLCM analysis.

The histological preparation of the osteochondral plugs (90 in total, 9 from each of 10 knees) as well as the assessment of osteoarthritic degeneration followed previously described protocols.^25^ For each plug, one histological section through the center of the plug was prepared (2.5 µm thickness). Briefly, deparaffinized tissue sections were stained with Safranin O and graded according to the Mankin Score^13^ by an experienced pathobiologist who was blinded to MR-derived measurements. Consecutive sections were stained with Picrosirius Red,^26,27^ followed by PLM at 200x magnification using a Axio Imager M2 with an MRc5 Axiocam camera and Zen blue software (Zeiss, Oberkochen, Germany). Following previously established protocols,^14^ images were recorded at 0°, 15°, 30°, 45°, 60°, 75°, and 90° polarizer pair positions. The corresponding background images were recorded for each angle after removal of the specimen from the microscope stage. To cover the full depth of the osteochondral plugs, adjacent images from the cartilage surface to the subchondral bone were recorded.

### Image Post-processing and Texture Analysis

#### MRI

**Figure 2.**
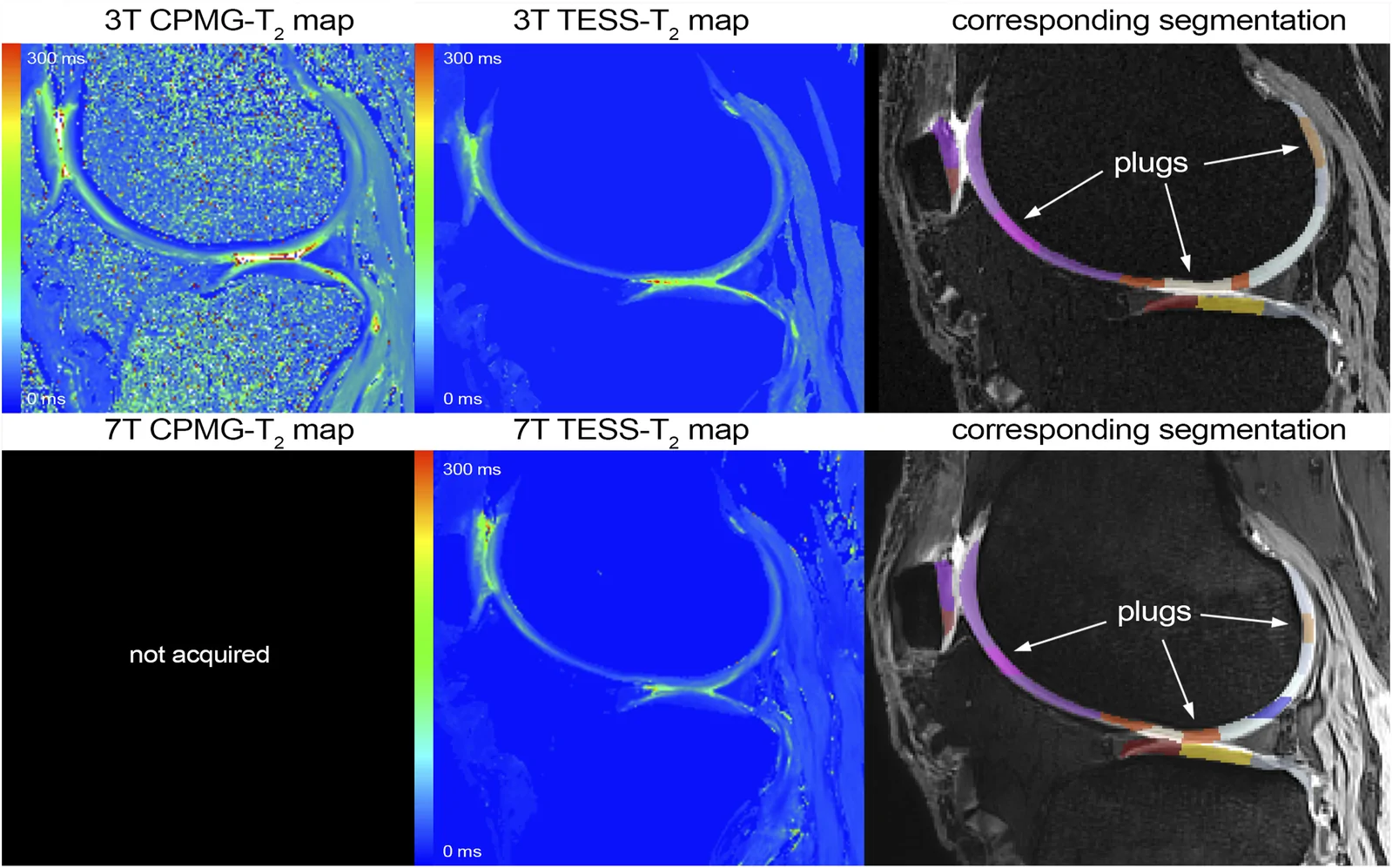
MESE-T₂ map and TESS-T₂ map acquired at both field strengths with the corresponding 3D-DESS images overlaid with an automated segmentation and manually selected plugs. MESE-T₂ is avoided at 7T due to excessive SAR, B1 inhomogeneity, and susceptibility-related signal issues. TESS - triple echo steady state; MESE - multi-slice multi-echo spin echo

The second echo of MESE and second echo of TESS sequence were registered to the DESS (combined from two echoes) using the B-spline registration in SimpleElastix implemented in Python 3.12 preserving the pixel intensities by avoiding unnecessary intensity normalization steps and by controlling the deformation regularization.^28^ As a similarity metric, advanced mattes mutual information was used, and an adaptive stochastic gradient descent as an optimizer. The obtained transformation parameters were then used to transform the respective T_2_ map.^29^ Visual inspection revealed insufficient registration quality (especially misplacement into the bone or synovial fluid) for the 3T-TESS image of donor 9 and the 3T-MESE images of donors 5 and 9 (in total, three datasets were omitted out of 30). As varying registration algorithms could alter pixel values, these datasets were excluded. The final plug ROIs were extracted using the automated segmentation and the registered T_2_ maps (Figure 2).

Mean T_2_ values were calculated ROI-wise, resulting in nine T_2_ values per knee for each of the three maps (7T TESS, 3T TESS, and 3T MESE). Only ROIs with more than fifty pixels were analyzed with GLCM. Slice-wise GLCM texture analysis was performed using an in-house-written script in MATLAB R2020a (version 9.8). ROIs were rotated (built-in MATLAB functions “regionprops” and “imrotate”), flattened and quantized into 16 gray levels and consecutive GLCM analysis was performed with an offset of 0° (direction parallel to the cartilage surface) and a step of length 1 (considering a pixel and its immediate neighbor), as used in Ref. 30. Using the GLCM_features1 function from the MATLAB Repository, twenty quantitative features were extracted (*autocorrelation, cluster prominence, cluster shade, contrast, correlation, difference entropy, difference variance, dissimilarity, energy, entropy, homogeneity, information measure, information measure of correlation 2, inverse difference moment normalized, inverse difference normalized (INN), maximum probability, sum average, sum entropy, sum of squares, sum variance*).^31^ For each analyzed ROI, GLCM feature values were averaged across slices.

### Polarized Light Microscopy

Pixel-by-pixel background correction was applied before computing the orientation and parallelism maps. Fibril orientation was derived from the Stokes parameters, which quantify alignment relative to the cartilage surface. The parallelism map reflects the degree of collagen fibril organization: a high parallelism index indicates well-aligned fibrils, while a low index suggests a disorganized or isotropic structure.^14^ The region-of-interest selected on the parallelism map included the whole cartilage from surface to deep cartilage zone – tidemark and subchondral bone was not considered. Parallelism value (arbitrary unit, ranging from 0 –low parallelism to 1 – high parallelism) was calculated as a mean value of two PLM slices.

### Statistical Analysis

All statistical analyses were performed in R version 4.4.1 (2024-06-14 ucrt). Data are described as mean ± standard deviation except for MS, which is described as median, minimum and maximum. To evaluate relationships between histology parameters, T_2_, and texture features, partial Spearman’s correlation coefficients were calculated, treating plug location as a confounding variable and removing its effect. Partial correlations were calculated for each T_2_ sequence separately, with each sequence considered as a separate family of tests and adjusted using the Benjamini–Hochberg correction. Variables significantly correlated with histology parameters were further analyzed using models with random intercepts.

As the MS is an ordinal variable, ranging from 0 (normal cartilage) to 14 (total cartilage loss), ordinal logistic regression with random intercepts was employed. To ensure adequate model fit and to satisfy assumptions, including the parallel odds assumption and low multicollinearity, the nine plug locations were pooled into three regions: medial condyle, trochlea, and lateral condyle. Values were not averaged, only samples locations were recategorized. Mankin scores were recategorized as follows:

0 – Normal cartilage
1–6 – Mild OA
7–10 – Moderate OA
11–14 – Severe OA

Models were fitted using the *clmm* function from the Ordinal package in R with MS as outcome variable. T_2_/texture features, T_2_ sequence and plug location were included as fixed effects, with a random intercept for each donor. The 3T (MESE) sequence and lateral condyle were set as the model reference levels. In case of high multicollinearity between fixed effects, nested models were compared using likelihood ratio tests by sequentially removing Modality and/or Zone and simpler model was chosen based on Akaike Information Criterion (AIC).

A similar approach was used for PLM orientation and parallelism, using linear mixed effects models with random intercepts, as these are continuous variables. Models were fitted using the *lmer* function from the lme4 package in R, with the T_2_/texture features, T_2_ sequence and plug location included as fixed effects and a random intercept for each donor. Models were estimated using restricted maximum likelihood (REML). Fixed-effect estimates and significance were calculated with degrees of freedom adjusted using the Kenward–Roger method.

Although interactions between T_2_/texture features, T_2_ sequence and plug location were of interest, the limited number of donor knees prevented reliable estimation of models including these interaction terms.

P-values lower than 0.05 were considered statistically significant.

## Results

**Figure 3.**
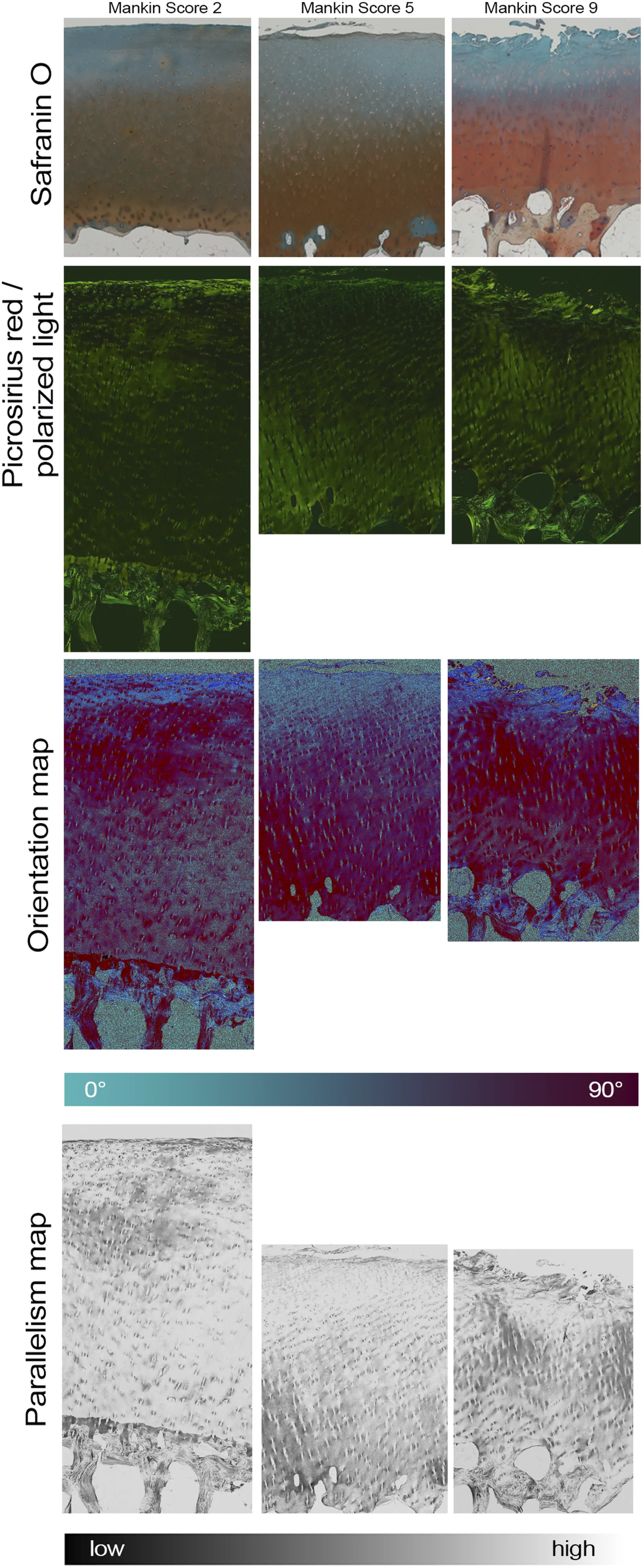
Representative images of different cartilage degeneration stages and their translation to the histological analysis

The Mankin Score of the extracted cartilage plugs ranged from 2 to 12 (higher values indicative of more severe cartilage degeneration) resulting in a median value of 5. The full descriptive statistics is listed in Supplementary Table. Examples for the histological analyses of three different cartilage degeneration stages are provided in Figure 3.

Significant positive correlations of weak-to-moderate magnitude were found between cartilage T_2_ values obtained from all three pulse sequences and the MS, which confirms the association of elevated T_2_ values and cartilage deterioration. Weak to moderate correlations were also observed between certain GLCM features and histological parameters. As such, the GLCM feature autocorrelation was negatively correlated with the MS (-0.63 for 7T TESS, -0.59 for 3T TESS and –0.43 for 3T MESE) and positively correlated with the PLM Orientation (0.53 for 7T TESS, 0.34 for 3T TESS and 0.34 for 3T MESE). The GLCM feature contrast, in turn, was positively correlated with the MS (0.61 for 7T TESS, 0.55 for 3T TESS and 0.44 for 3T MESE) and negatively correlated with the PLM Orientation ( -0.33 for 3T TESS). Notably, the highest number of significant correlations was between features calculated from 3T TESS maps and MS. All correlations are listed in Table 2.

**Table 2.** Partial Spearman’s Correlation Coefficients Between Histology Parameters, T_2_, and Texture Features, With Plug Location Treated as a Confounding Variable. TP-Values Were Adjusted Using the Benjamini-Hochberg Procedure

Field strength (T_2_ method)	Histology								
	Mankin score [a.u.]			Orientation (PLM) [degrees]			Parallelism (PLM) [a.u.]		
	7T (TESS)	3T (TESS)	3T (MESE)	7T (TESS)	3T (TESS)	3T (MESE)	7T (TESS)	3T (TESS)	3T (MESE)
**Mean T**_**2**_ **[ms]**	0.33 (0.14, 0.52) *	0.46 (0.25, 0.64) ***	0.48 (0.28, 0.65) **	-0.09 (-0.28, 0.13)	0.06 (-0.16, 0.27)	0.12 (-0.10, 0.33)	-0.12 (-0.31, 0.08)	0.06 (-0.15, 0.26)	-0.02 (-0.25, 0.22)
**Texture features from T**_**2**_ **maps [all a.u.]:**									
Autocorrelation	-0.63 (-0.75, -0.46) ***	-0.59 (-0.73, -0.41) ***	-0.43 (-0.62, -0.22) **	0.53 (0.37, 0.66) ***	0.43 (0.22, 0.61) ***	0.34 (0.13, 0.54) *	0.21 (-0.01, 0.40)	0.26 (0.02, 0.47)	0.25 (0.01, 0.46)
Contrast	0.61 (0.42, 0.74) ***	0.55 (0.38, 0.69) ***	0.44 (0.19, 0.67) **	-0.33 (-0.49, -0.14) *	-0.27 (-0.47, -0.05)	-0.12 (-0.32, 0.10)	-0.11 (-0.33, 0.09)	-0.14 (-0.35, 0.10)	-0.17 (-0.39, 0.07)
Correlation	-0.23 (-0.45, -0.03)	-0.53 (-0.67, -0.34) ***	-0.30 (-0.53, -0.05)	0.23 (0.02, 0.43)	0.13 (-0.07, 0.33)	0.16 (-0.04, 0.36)	0.12 (-0.11, 0.31)	0.05 (-0.16, 0.27)	0.07 (-0.17, 0.29)
Cluster Prominence	-0.03 (-0.24, 0.18)	-0.18 (-0.42, 0.04)	-0.28 (-0.49, -0.04)	-0.02 (-0.23, 0.17)	-0.13 (-0.34, 0.10)	0.08 (-0.15, 0.31)	-0.05 (-0.27, 0.20)	-0.13 (-0.35, 0.09)	-0.06 (-0.29, 0.18)
Cluster Shade	-0.06 (-0.26, 0.16)	0.07 (-0.17, 0.29)	0.01 (-0.23, 0.23)	-0.12 (-0.33, 0.10)	-0.11 (-0.34, 0.13)	-0.11 (-0.36, 0.16)	-0.06 (-0.26, 0.14)	0.08 (-0.15, 0.32)	-0.03 (-0.30, 0.21)
Dissimilarity	0.24 (0.03, 0.44)	0.13 (-0.12, 0.36)	0.18 (-0.09, 0.43)	-0.22 (-0.41, -0.01)	-0.09 (-0.32, 0.13)	-0.08 (-0.28, 0.12)	-0.11 (-0.31, 0.10)	-0.10 (-0.32, 0.13)	-0.07 (-0.29, 0.18)
Energy	-0.09 (-0.31, 0.13)	-0.02 (-0.24, 0.22)	-0.04 (-0.31, 0.22)	0.17 (-0.05, 0.37)	0.04 (-0.20, 0.27)	-0.13 (-0.36, 0.11)	0.17 (-0.06, 0.37)	0.14 (-0.08, 0.37)	0.08 (-0.15, 0.32)
Entropy	0.49 (0.29, 0.66) ***	0.50 (0.28, 0.67) ***	0.42 (0.18, 0.63) **	-0.16 (-0.37, 0.05)	-0.26 (-0.45, -0.05)	0.05 (-0.19, 0.29)	-0.08 (-0.31, 0.15)	-0.12 (-0.34, 0.11)	-0.00 (-0.22, 0.23)
Homogeneity	-0.23 (-0.43, 0.01)	-0.12 (-0.36, 0.10)	-0.17 (-0.41, 0.08)	0.23 (0.02, 0.41)	0.11 (-0.09, 0.30)	0.06 (-0.17, 0.26)	0.09 (-0.13, 0.27)	0.08 (-0.15, 0.30)	0.07 (-0.15, 0.31)
Maximum probability	-0.09 (-0.32, 0.13)	-0.07 (-0.32, 0.16)	-0.04 (-0.29, 0.22)	0.17 (-0.06, 0.37)	0.12 (-0.11, 0.35)	-0.12 (-0.34, 0.10)	0.14 (-0.08, 0.33)	0.16 (-0.08, 0.37)	0.09 (-0.14, 0.32)
Sum of squares	-0.02 (-0.24, 0.20)	-0.00 (-0.25, 0.23)	-0.06 (-0.31, 0.18)	0.19 (-0.03, 0.38)	0.14 (-0.09, 0.35)	0.09 (-0.18, 0.33)	0.17 (-0.06, 0.37)	0.08 (-0.17, 0.32)	0.11 (-0.14, 0.36)
Sum average	-0.02 (-0.24, 0.20)	0.03 (-0.21, 0.27)	-0.03 (-0.26, 0.24)	0.18 (-0.03, 0.37)	0.12 (-0.09, 0.34)	0.08 (-0.16, 0.33)	0.17 (-0.06, 0.37)	0.07 (-0.18, 0.32)	0.12 (-0.13, 0.36)
Sum variance	-0.02 (-0.23, 0.20)	0.01 (-0.20, 0.25)	-0.05 (-0.29, 0.21)	0.19 (-0.02, 0.40)	0.12 (-0.12, 0.35)	0.09 (-0.17, 0.34)	0.18 (-0.06, 0.38)	0.07 (-0.17, 0.31)	0.11 (-0.15, 0.35)
Sum entropy	-0.17 (-0.37, 0.02)	-0.54 (-0.71, -0.33) ***	-0.23 (-0.49, 0.03)	0.01 (-0.21, 0.21)	0.14 (-0.09, 0.35)	0.15 (-0.08, 0.37)	-0.04 (-0.25, 0.18)	-0.03 (-0.28, 0.20)	-0.16 (-0.40, 0.08)
Difference variance	0.24 (0.03, 0.43)	0.07 (-0.17, 0.33)	0.21 (-0.05, 0.45)	-0.25 (-0.45, -0.04)	-0.06 (-0.27, 0.16)	-0.07 (-0.28, 0.14)	-0.11 (-0.32, 0.12)	-0.12 (-0.33, 0.11)	-0.01 (-0.24, 0.24)
Difference entropy	0.24 (0.02, 0.45)	0.15 (-0.10, 0.38)	0.20 (-0.06, 0.42)	-0.21 (-0.40, -0.01)	-0.08 (-0.31, 0.13)	-0.07 (-0.28, 0.15)	-0.14 (-0.33, 0.08)	-0.11 (-0.33, 0.12)	-0.11 (-0.36, 0.12)
Information measure	0.12 (-0.10, 0.33)	0.06 (-0.19, 0.28)	0.20 (-0.05, 0.42)	-0.02 (-0.21, 0.19)	0.14 (-0.09, 0.35)	-0.07 (-0.31, 0.15)	-0.12 (-0.33, 0.11)	0.06 (-0.19, 0.30)	-0.07 (-0.28, 0.15)
Information measure of correlation 2	-0.12 (-0.33, 0.08)	-0.10 (-0.31, 0.15)	-0.27 (-0.47, -0.04)	0.01 (-0.17, 0.20)	-0.15 (-0.37, 0.08)	0.11 (-0.11, 0.34)	0.10 (-0.11, 0.32)	-0.10 (-0.31, 0.13)	-0.01 (-0.21, 0.20)
Inverse difference normalized (INN)	-0.47 (-0.62, -0.28) ***	-0.30 (-0.52, -0.08) *	-0.17 (-0.43, 0.09)	0.29 (0.08, 0.47) *	0.10 (-0.12, 0.30)	0.06 (-0.14, 0.25)	0.12 (-0.10, 0.32)	-0.00 (-0.23, 0.22)	0.08 (-0.16, 0.32)
Inverse difference moment normalized	-0.23 (-0.44, -0.02)	-0.13 (-0.36, 0.12)	-0.21 (-0.47, 0.06)	0.23 (0.03, 0.43)	0.09 (-0.14, 0.30)	0.07 (-0.14, 0.27)	0.10 (-0.12, 0.31)	0.11 (-0.12, 0.33)	0.03 (-0.20, 0.27)

PLM – polarized light microscopy; TESS - tripple echo steady state; MESE - multi-slice multi-echo spin echo.

***p < 0.001, **0.001 ≤ p < 0.01, *0.01 ≤ p < 0.05, • 0.05 ≤ p < 0.06, (blank) p ≥ 0.06.

7T (TESS) N = 10.

3T (TESS) N = 9.

3T (MESE) N = 8.

### Models

Ordinal logistic regression revealed that T_2_ relaxation time was a significant predictor of Mankin scores, with each 1 ms increase in T_2_ associated with a 8.2% increase in the odds of reaching a higher histological grade (p < 0.001). While the relationship between T_2_ relaxation times and Mankin score remained consistent across modalities, the use of 3T (TESS) and 7T (TESS) sequences in comparison to the standard 3T (MESE) significantly increased the baseline odds of identifying higher grades by 1,556% (p < 0.001) and 1,778% (p < 0.001) respectively. Additionally, the medial condyle demonstrated significantly higher odds of advanced degeneration (160.1%, p = 0.028) relative to the lateral condyle when controlling for T_2_ values and imaging modality, which corresponds to clinical experience.

For autocorrelation, contrast, and correlation, the imaging modality used for the T_2_ map did not significantly affect the odds of a higher MS. A one a.u. increase in autocorrelation and correlation was associated with a decrease in the odds of more severe cartilage degeneration by 6.7% (p < 0.001) and 98.6% (p < 0.001), respectively, while a one a.u. increase in contrast was associated with a 45.1% increase in those odds.

Due to high multicollinearity with imaging modality, entropy, sum entropy, and INN were each analyzed using simpler models (Table 3). A one-unit increase in entropy was associated with an 8,495.1% increase in the odds of advanced degeneration, whereas sum entropy and INN were associated with decreases of 98.6% (p < 0.001) and 99.99% (p < 0.001), respectively.

**Table 3.** Ordinal Logistic Regression Results. in Case of High Multicollinearity Between Fixed Effects, a Simpler Model was Chosen Based on Akaike Information Criterion (AIC)

Ordinal logistic regression with random intercept					
Model	Variable	OR	Lower CI	Upper CI	p-value
Mankin score ∼ T_2_ [ms]	Mean T_2_	1.082	1.048	1.117	<0.001
	3T (TESS)	16.665	3.737	74.32	<0.001
	7T (TESS)	18.78	3.926	89.84	<0.001
	Zone Trochlea	1.883	0.791	4.483	0.153
	Zone Medial condyle	2.601	1.108	6.104	0.028
Mankin score ∼ Autocorrelation	Autocorrelation	0.933	0.914	0.952	<0.001
	3T (TESS)	2.289	0.871	6.017	0.093
	7T (TESS)	1.727	0.665	4.486	0.262
	Zone Trochlea	1.271	0.485	3.331	0.626
	Zone Medial condyle	3.873	1.466	10.231	0.006
Mankin score ∼ Contrast	Contrast	1.451	1.286	1.636	<0.001
	3T (TESS)	1.192	0.49	2.895	0.699
	7T (TESS)	1.03	0.424	2.502	0.948
	Zone Trochlea	1.297	0.517	3.255	0.580
	Zone Medial condyle	2.085	0.826	5.265	0.120
Mankin score ∼ Correlation	Correlation	0.014	0.002	0.109	<0.001
	3T (TESS)	1.155	0.509	2.62	0.730
	7T (TESS)	1.005	0.446	2.264	0.991
	Zone Trochlea	1.692	0.724	3.956	0.225
	Zone Medial condyle	2.691	1.159	6.248	0.021
Mankin score ∼ Entropy	Entropy	85.951	15.634	472.527	<0.001
	Zone Trochlea	0.967	0.392	2.387	0.942
	Zone Medial condyle	3.061	1.292	7.255	0.011
Mankin score ∼ Sum Entropy	Sum Entropy	0.014	0.002	0.101	<0.001
	Zone Trochlea	2.733	1.139	6.56	0.024
	Zone Medial condyle	3.316	1.434	7.673	0.005
Mankin score ∼ INN	INN	3.606E-10	8.041E-15	1.618E-05	<0.001
	Zone Trochlea	1.59	0.68	3.73	0.288
	Zone Medial condyle	2.63	1.12	6.15	0.026

OR – odds ratio, CI – confidence interval, INN - Inverse difference normalized.

Baseline of the model is set as values calculated from the 3T (MESE) sequence in the lateral femoral condyle.

Mankin scores were pooled in the following categories: 0 – Normal cartilage, 1 – 6 Mild OA, 7 – 10 Moderate OA, 11 – 14 Severe OA.

Finally, after controlling for feature values and imaging modality, the medial condyle showed significantly higher odds of advanced degeneration compared to the lateral condyle across all features except contrast. The trochlea showed significantly higher odds of advanced degeneration only for sum entropy (p = 0.024).

Based on significant correlations with the PLM orientation, only features autocorrelation, contrast and INN were analyzed using mixed-effects multiple linear regression. In these models, neither plug location nor imaging modality had a significant effect. A one-unit increase in autocorrelation was associated with a 0.045° increase in PLM orientation (p < 0.001), while a one-unit increase in contrast was associated with a 0.163° decrease (p < 0.001). Effect of INN was not significant. All models are listed in Tables 3 and 4.

**Table 4.** Result of Linear Mixes Effects Models With Random Intercepts. Only Variables Significantly Correlated With PLM Orientation Were Analyzed

Linear mixed-effects with random intercept					
Model	Variable	Estimate	Lower CI	Upper CI	p-value
PLM Orientation ∼ Autocorrelation	Autocorrelation	0.045	0.035	0.055	<0.001
	3T (TESS)	-0.309	-0.963	0.344	0.352
	7T (TESS)	-0.361	-1.016	0.293	0.278
	Zone Trochlea	0.411	-0.221	1.043	0.202
	Zone Medial condyle	-0.563	-1.186	0.060	0.076
PLM Orientation ∼ Contrast	Contrast	-0.163	-0.251	-0.075	<0.001
	3T (TESS)	-0.057	-0.787	0.673	0.878
	7T (TESS)	-0.005	-0.734	0.724	0.989
	Zone Trochlea	0.197	-0.519	0.913	0.588
	Zone Medial condyle	-0.380	-1.103	0.343	0.301
PLM Orientation ∼ INN	INN	8.499	-0.882	17.880	0.076
	3T (TESS)	-0.019	-0.765	0.727	0.960
	7T (TESS)	-0.011	-0.756	0.734	0.977
	Zone Trochlea	0.027	-0.699	0.752	0.942
	Zone Medial condyle	-0.607	-1.332	0.118	0.101

CI – confidence interval, INN - Inverse difference normalized.

Baseline of the model is set as values calculated from the 3T (MESE) sequence in the lateral femoral condyle.

## Discussion

Texture features derived from quantitative MRI maps of cartilage have attracted increasing attention from the OA community in recent years. To the authors’ knowledge, this is the first ex vivo study to attempt to validate individual features of texture analysis of T_2_ maps using the histological gold standard. Six out of the twenty features (autocorrelation, contrast, correlation, entropy, sum entropy and inverse difference moment normalized) were found to correlate with the MS; autocorrelation, contrast and entropy were also found to be correlated with collagen orientation. The demonstrated correlations between image-derived features and histological scores of cartilage represent a decisive step toward in vivo monitoring of cartilage degeneration and potentially regeneration and could help in identifying therapies that restore articular cartilage quantity and quality.

This is the first study investigating the relationship between cartilage texture features extracted from T_2_ maps and histological analyses. Automated segmentation provided a reproducible way to segment the whole (femoral) cartilage and allowed co-localization of the plugs corresponding to the histologically targeted tissue. The main findings of this study are: a) some texture features extracted from T_2_ maps are correlated to the histologically assessed stage of cartilage degeneration and collagen fiber orientation, b) several correlations were observed across the two field strengths and acquisition methods (TESS at 3T and 7T; MESE at 3T), namely the correlation of the GLCM features autocorrelation, contrast and correlation with the MS and autocorrelation with fiber orientation. To draw the connection between GLCM features and the histological parameters, multiple aspects must be taken into consideration. Based on their mathematical definitions, some GLCM features are highly intercorrelated and can be divided into three categories: 1) measures of gray level homogeneity (e.g. homogeneity, energy, sum average, inverse difference moment normalized), 2) measures of presence of edges or disorderliness (e.g. contrast, dissimilarity, entropy, difference variance, difference entropy) and 3) probability of occurrence of specific gray level pairs (e.g. autocorrelation, and the GLCM correlation feature).^32,33^ Homogeneity characterizes the distribution of gray levels within the region of interest (ROI). Higher homogeneity indicates more uniform gray values, while lower homogeneity signals greater variation and a grainier texture within the ROI. In this study, lower homogeneity features were associated with higher MS scores, and therefore higher severity of OA lesions. Carballido-Gamio et al. reported similar results – homogeneity calculated in the direction parallel to the bone-cartilage interface was lower in OA patients.^34^

A high contrast value signifies large differences in gray levels between neighboring pixels, whereas a low contrast value indicates that adjacent pixels have similar gray levels. Features of texture disorderliness (contrast and entropy) were positively correlated with the MS and were negatively correlated with collagen orientation. The higher the disorderliness, the higher the degree of cartilage degeneration and the lower the orientation angle of collagen fibrils in relation to cartilage surface. Joseph et al. observed contrast being elevated in those at risk for OA, Carballido-Gamio et al. demonstrated elevated contrast in patients with mild OA and Blumenkrantz et al. showed elevated entropy in OA patients.^11,34,35^

Autocorrelation and the GLCM correlation feature indicate the extent of repeating patterns within an image, with higher values signifying more organized, repetitive textures, and lower values suggesting greater randomness. Our study revealed the correlation of an increase in repeating patterns in cartilage with lower MS and higher orientation angle. Peuna et al. observed higher autocorrelation values associated with OA, while the GLCM feature correlation was lower. They also found increased values of features tied to disorderliness and contrast and lower values of features tied to homogeneity.^12^ Apart from elevated T_2_ values being a sign of cartilage matrix degradation and an increase in water mobility,^36^ our results suggest the higher the disorderliness and the lower the homogeneity, the higher MS grade. A possible explanation is that cartilage degeneration disrupts the collagen network and increases microstructural heterogeneity. As a result, neighboring pixels become less similar, which reduces GLCM homogeneity and increases measures of randomness. Degeneration may also disturb the normal zonal organization of cartilage, leading to localized regions with different structural and compositional properties that appear as a more irregular texture pattern. This results are in agreement with the body of literature.^11,34,35^ This interpretation is consistent with our findings, as the texture analysis was compared to histology.

Interestingly, texture features derived from T_2_ maps did not correlate with collagen parallelism assessed by polarized light microscopy, although both reflect the collagen content and orientation. This can be possible attributed to the different spatial resolution of these two modalities as well as to the fact that texture features reflect compositional heterogeneity, whereas PLM captures architectural alignment (measures local collagen organization).

The direction of the partial correlations between MS, T_2_ and texture features were confirmed by the modelling approach. After adjusting for measurement sequence and cartilage plug location, higher T_2_ was significantly associated with worse histological cartilage degeneration. Since each sequence measures different absolute T_2_ values (Table 2), and the effect of sequence was significant, it is therefore possible that the relationship between T_2_ and Mankin score differs between the 3T MESE, 3T TESS and 7T TESS sequences, whereas the relationship between 3T TESS and 7T TESS is comparable. Given the sample size constraints, we were unable to test for a sequence × T_2_ interaction. The model assumes that the relationship between T_2_ and Mankin score is the same across all sequences (additivity on the log-odds scale), which should be interpreted with caution. In contrast, texture features (autocorrelation, contrast, and correlation) showed no significant sequence effects in predicting Mankin score, suggesting that texture analysis yields consistent results regardless of mapping sequence or field strength. Entropy, sum entropy, and INN showed high collinearity with the acquisition sequence, suggesting these texture features reflect both tissue microstructure and acquisition-related differences. Interpretation of their relationship with cartilage degeneration requires careful consideration of how the data were acquired. The models further showed that degeneration was more severe in the medial condyle, which is the weight-bearing region. This is consistent with the body of literature showing that OA severity is greater in medial weight-bearing areas of the femoral cartilage.^37,38^ For the texture feature sum entropy, the trochlear region showed higher odds of worse MS in addition to the medial condyle region. However, as this was one of the models with high collinearity, this results should be interpreted with caution.

Although T_2_ was not significantly correlated with PLM parameters, some texture features did correlate. In the mixed-effects multiple linear regression, the features not only showed a correlation with collagen fiber orientation, but again no significant effects of sequence or plug location, further suggesting that T_2_ values texture is closely related to the cartilage structure.

This study has certain limitations. First and foremost, this study was designed as an ex vivo study to allow for a histological analysis. Hence, potential sources of additional variability, such as motion artifacts when performed in patients were not accounted for. The only alternative study design which would combine in vivo imaging and allow for histological assessment of cartilage, would be to include patients, who undergo subsequent joint replacement. However, in joint replacement the cuts are defined by the cutting guide and would not have aligned with the predefined nine regions in which MRChondralHealth subdivides the femur and thus would not have allowed for the standardized harvest of osteochondral plugs from the exact same nine regions of the MRChondralHealth software. Second, even though the number of samples was relatively low, it was in line with many ex-vivo studies given the scarcity of fresh non-frozen body donors with sufficiently good cartilage that provides a good range of different stages of degeneration. In addition, we tested multiple samples per donor. Variations in joint positioning between scans or subjects, including magic angle effect, may introduce non-biological heterogeneity in texture metrics, potentially confounding the interpretation of texture features. The lack of completely healthy cartilage plugs may be also a limiting factor, but unfortunately the study design as such (donor knees) did not allow for harvesting healthy tissue. Considering the study’s exploratory, hypothesis-generating design, we regard this limitation as acceptable. Although the modelling results are promising, they should be interpreted with caution. The nested data structure, with multiple plugs and repeated measurements per donor, required the use of complex statistical models. However, the small data imposed limitations on how relationships could be modelled, while the underlying model assumptions could be satisfied. Since not all cartilage locations spanned the full range of Mankin scores, and nine plug locations proved excessive for reliable parameter estimation, both Mankin scores and cartilage locations required recategorization. Additionally, the large odds ratios and wide confidence intervals observed in some models indicate insufficient statistical power, suggesting that the true effects were likely small in magnitude. A larger sample size would be needed to reliably estimate interaction effects, which are of interest given the nature of the data.

## Conclusion

In conclusion, texture features extracted from quantitative MRI images of articular cartilage provide additional information about collagen content and orientation associated with the tissue’s degeneration status. Texture analysis could therefore be used prospectively to monitor OA patients undergoing either conservative or surgical treatment. The correlations between image-derived features with histological quality scores demonstrated here represent a decisive step toward in vivo cartilage evaluation, and could help in the development or assessment of therapies aimed at restoring cartilage quantity and quality.

## Supplemental Material

Supplemental Material - Textural Features from Quantitative MRI Using T2 Relaxation Times Correlate with Histological Cartilage Degeneration: An Ex-Vivo Study Supplemental Material for Textural Features from Quantitative MRI Using T2 Relaxation Times Correlate with Histological Cartilage Degeneration: An Ex-Vivo Study by Markus Schreiner, Stefan Toegel, Veronika Janacova, Pavol Szomolanyi, Didier Laurent, Franziska Saxer, Matthias Schieker, Rahel Heule, Oliver Bieri, Esther Raithel, Christoph Fuchssteiner, Wolfgang J. Weninger, Reinhard Windhager, Siegfried Trattnig, Benedikt Hager, Vladimir Juras in CARTILAGE

## Data Availability

Data cannot be shared due to ethical, legal, or commercial restrictions.*
