# Supplementary material for: Textural Features from Quantitative MRI Using T2 Relaxation Times Correlate with Histological Cartilage Degeneration: An Ex-Vivo Study
Source: Cartilage. 2026 Aug 20:19476035261478556. Online ahead of print. doi: 10.1177/19476035261478556 (PMC13493633; doi:10.1177/19476035261478556)
Supplement: Supplementary file 1 [file sj-pdf-1-car-10.1177_19476035261478556.pdf]

Table 2. Descriptive Statistics. Variables are Described as Mean ± Sd, Except for Mankin Score, Which is Described as Median (Min, Max)

<!--Col Count:26-->T_2 method	Label	Mankin score [a.u.]	Orientation (PLM) [degrees]	Parallelism (PLM) [a.u.]	Mean T_2 [ms]	Autocorrelation	Contrast	Correlation	Cluster prominence	Cluster shade	Dissimilarity	Energy	Entropy	Homogeneity	Maximum probability	Sum of squares	Sum average	Sum variance	Sum entropy	Difference variance	Difference entropy	Information measure of correlation	Information measure of correlation 2	Inverse difference normalized INN	Inverse difference moment normalized
3T CPMG	femoral medial posterior	5 (3, 7)	35.58 ± 2.50	0.64 ± 0.05	93.24 ± 20.24	101.89 ± 24.77	10.50 ± 7.22	0.49 ± 0.21	2664.27 ± 1345.59	-62.04 ± 28.24	2.55 ± 0.90	0.03 ± 0.01	3.61 ± 0.15	0.42 ± 0.08	0.08 ± 0.03	116.95 ± 15.95	20.37 ± 1.78	348.30 ± 60.19	2.80 ± 0.16	11.75 ± 7.42	1.87 ± 0.22	-0.42 ± 0.05	0.92 ± 0.03	0.87 ± 0.04	0.96 ± 0.03
3T CPMG	femoral medial central	6 (2, 8)	33.75 ± 3.37	0.61 ± 0.11	88.21 ± 9.96	79.94 ± 32.07	10.05 ± 6.52	0.48 ± 0.11	3280.54 ± 481.26	-43.98 ± 110.80	2.71 ± 0.51	0.03 ± 0.00	3.68 ± 0.12	0.43 ± 0.04	0.08 ± 0.01	103.95 ± 30.67	18.80 ± 3.64	303.81 ± 104.91	2.86 ± 0.07	13.30 ± 4.53	1.97 ± 0.11	-0.42 ± 0.04	0.93 ± 0.02	0.86 ± 0.02	0.95 ± 0.02
3T CPMG	femoral medial anterior	4 (2, 8)	35.14 ± 2.16	0.64 ± 0.10	64.65 ± 18.47	67.21 ± 30.80	4.77 ± 2.37	0.84 ± 0.06	7530.90 ± 2486.53	232.92 ± 126.27	1.08 ± 0.33	0.08 ± 0.02	3.21 ± 0.29	0.70 ± 0.06	0.20 ± 0.05	46.33 ± 17.08	11.01 ± 2.29	130.64 ± 53.81	2.71 ± 0.15	4.77 ± 2.37	1.26 ± 0.20	-0.61 ± 0.05	0.96 ± 0.01	0.94 ± 0.02	0.98 ± 0.01
3T CP	trochl	5 (4,	34.6	0.5	79.	75.67	8.0	0.53	2319.28	-14	2.14	0.04	3.62	0.48	0.0	84.6	17.0	244.9	2.76	8.9	1.7	-0.46	0.93	0.89	0.97

<!--Col Count:26-->T_2 method	Label	Mankin score [a.u.]	Orientation (PLM) [degrees]	Parallelism (PLM) [a.u.]	Mean T_2 [ms]	Autocorrelation	Contrast	Correlation	Cluster prominence	Cluster shade	Dissimilarity	Energy	Entropy	Homogeneity	Maximum probability	Sum of squares	Sum average	Sum variance	Sum entropy	Difference variance	Difference entropy	Information measure of correlation	Information measure of correlation 2	Inverse difference normalized INN	Inverse difference moment normalized
MG	ear medial	8)	3 ± 2.11	8 ± 0.10	79 ± 25.20	± 19.11	9 ± 2.84	± 0.11	± 943.82	.99 ± 66.16	± 0.45	± 0.01	± 0.18	± 0.05	9 ± 0.02	6 ± 21.65	8 ± 2.53	0 ± 74.54	± 0.15	7 ± 3.15	6 ± 0.17	± 0.04	± 0.02	± 0.02	± 0.01
3T CPMG	trochlear central	5.5 (4,9)	33.70 ± 2.14	0.55 ± 0.14	67.99 ± 16.42	53.69 ± 19.21	6.70 ± 2.28	0.69 ± 0.07	3783.50 ± 1073.79	119.10 ± 93.67	1.61 ± 0.31	0.04 ± 0.01	3.63 ± 0.15	0.56 ± 0.05	0.09 ± 0.02	50.56 ± 15.92	12.33 ± 2.50	132.96 ± 52.18	2.87 ± 0.07	6.20 ± 1.93	1.62 ± 0.15	-0.43 ± 0.04	0.92 ± 0.02	0.91 ± 0.02	0.98 ± 0.01
3T CPMG	trochlear lateral	4.5 (3,7)	36.68 ± 3.69	0.58 ± 0.13	69.86 ± 20.64	91.84 ± 27.56	7.26 ± 1.62	0.68 ± 0.09	3955.22 ± 1690.79	-80.75 ± 66.78	1.98 ± 0.25	0.03 ± 0.00	3.73 ± 0.14	0.50 ± 0.04	0.08 ± 0.01	106.25 ± 14.62	19.14 ± 1.62	311.93 ± 51.72	2.92 ± 0.13	7.64 ± 1.81	1.76 ± 0.10	-0.43 ± 0.05	0.93 ± 0.02	0.89 ± 0.01	0.97 ± 0.01
3T CPMG	femoral lateral anteri	5 (3,9)	35.55 ± 3.12	0.62 ± 0.07	64.58 ± 9.43	65.66 ± 21.73	4.46 ± 1.74	0.82 ± 0.09	5536.89 ± 1349.57	128.50 ± 124.51	1.08 ± 0.31	0.05 ± 0.01	3.38 ± 0.27	0.69 ± 0.07	0.13 ± 0.03	51.60 ± 17.11	12.29 ± 2.43	144.33 ± 54.10	2.86 ± 0.12	4.46 ± 1.74	1.29 ± 0.19	-0.57 ± 0.06	0.96 ± 0.01	0.94 ± 0.02	0.98 ± 0.01

<!-- Col Count:26 -->T_2 method	Label	Manikin score [a.u.]	Orientation (PLM) [degrees]	Parallelism (PLM) [a.u.]	Mean T_2 [ms]	Autocorrelation	Contrast	Correlation	Cluster prominence	Cluster shade	Dissimilarity	Energy	Entropy	Homogeneity	Maximum probability	Sum of squares	Sum average	Sum variance	Sum entropy	Difference variance	Difference entropy	Information measure of correlation	Information measure of correlation 2	Inverse difference normalized INN	Inverse difference moment normalized
	or																								
3T CPMG	femoral lateral central	5 (4, 7)	34.31 ± 1.91	0.61 ± 0.08	85.80 ± 21.83	93.03 ± 20.34	7.35 ± 3.93	0.58 ± 0.14	1995.00 ± 967.44	-39.15 ± 64.93	1.94 ± 0.56	0.03 ± 0.01	3.63 ± 0.18	0.50 ± 0.07	0.08 ± 0.03	101.94 ± 20.77	19.26 ± 2.09	305.24 ± 68.83	2.77 ± 0.14	7.35 ± 3.93	1.72 ± 0.19	-0.38 ± 0.04	0.90 ± 0.03	0.90 ± 0.03	0.97 ± 0.01
3T CPMG	femoral lateral posterior	4.5 (2, 8)	35.66 ± 3.06	0.66 ± 0.06	64.52 ± 27.22	103.91 ± 23.56	5.47 ± 1.95	0.67 ± 0.18	2011.71 ± 935.88	-36.29 ± 42.09	1.61 ± 0.33	0.04 ± 0.01	3.58 ± 0.22	0.53 ± 0.07	0.09 ± 0.02	111.72 ± 19.68	20.09 ± 2.15	337.88 ± 64.16	2.79 ± 0.13	5.47 ± 1.95	1.53 ± 0.12	-0.45 ± 0.07	0.93 ± 0.03	0.91 ± 0.02	0.98 ± 0.01
3T TESS	femoral medial posterior	5 (3, 12)	35.34 ± 2.44	0.62 ± 0.07	53.18 ± 9.01	94.82 ± 25.98	8.88 ± 3.66	0.57 ± 0.14	2151.67 ± 976.16	-57.64 ± 54.31	2.16 ± 0.47	0.03 ± 0.00	3.70 ± 0.23	0.45 ± 0.06	0.07 ± 0.01	107.98 ± 17.26	19.71 ± 1.73	320.06 ± 59.17	2.80 ± 0.15	7.93 ± 3.77	1.76 ± 0.15	-0.41 ± 0.04	0.92 ± 0.02	0.89 ± 0.03	0.97 ± 0.01
3T TESS	femoral media	6 (2, 8)	33.01 ± 3.85	0.61 ± 0.10	55.83 ±	86.37 ± 18.96	9.30 ± 3.72	0.60 ± 0.15	2741.68 ± 514.	-43.07 ±	2.29 ± 0.48	0.03 ± 0.	3.74 ± 0.	0.45 ± 0.05	0.06 ± 0.01	103.15 ± 13	19.09 ± 1.	301.41 ± 48.	2.72 ± 0.	8.95 ± 4.03	1.84 ± 0.16	-0.40 ± 0.03	0.92 ± 0.01	0.88 ± 0.02	0.96 ± 0.01

<!-- Col Count:26 -->T_2 method	Label	Mankin score [a.u.]	Orientation (PLM) [degrees]	Parallelism (PLM) [a.u.]	Mean T_2 [ms]	Autocorrelation	Contrast	Correlation	Cluster prominence	Cluster shade	Dissimilarity	Energy	Entropy	Homogeneity	Maximum probability	Sum of squares	Sum average	Sum variance	Sum entropy	Difference variance	Difference entropy	Information measure of correlation	Information measure of correlation 2	Inverse difference normalized INN	Inverse difference moment normalized
	l central				7.99				97	57.06		00	26			.61	53	44	29						
3T TESS	femoral medial anterior	4 (2, 8)	33.53 ± 5.25	0.61 ± 0.13	43.33 ± 12.22	80.16 ± 33.47	5.03 ± 2.68	0.73 ± 0.19	5477.19 ± 1971.25	144.62 ± 61.90	0.95 ± 0.19	0.05 ± 0.01	3.57 ± 0.25	0.69 ± 0.04	0.13 ± 0.02	56.49 ± 11.99	13.12 ± 1.49	157.90 ± 38.71	2.79 ± 0.24	3.03 ± 1.20	1.24 ± 0.12	-0.58 ± 0.03	0.97 ± 0.01	0.94 ± 0.02	0.99 ± 0.00
3T TESS	trochlear medial	5 (4, 9)	34.30 ± 2.21	0.58 ± 0.09	45.34 ± 8.62	77.26 ± 17.50	9.16 ± 2.68	0.57 ± 0.17	2469.02 ± 1468.29	-25.54 ± 25.57	2.37 ± 0.50	0.03 ± 0.01	3.79 ± 0.15	0.44 ± 0.04	0.06 ± 0.01	98.13 ± 13.48	18.67 ± 1.83	289.40 ± 55.40	2.75 ± 0.23	10.16 ± 4.33	1.84 ± 0.18	-0.46 ± 0.03	0.94 ± 0.02	0.88 ± 0.02	0.96 ± 0.01
3T TESS	trochlear central	5 (3, 9)	33.81 ± 2.03	0.53 ± 0.15	42.33 ± 7.03	75.50 ± 19.56	6.56 ± 2.80	0.71 ± 0.17	3534.59 ± 1083.00	4.62 ± 62.85	1.70 ± 0.33	0.03 ± 0.00	3.75 ± 0.23	0.53 ± 0.04	0.07 ± 0.01	78.83 ± 16.23	16.22 ± 2.04	219.25 ± 56.98	3.08 ± 0.35	6.45 ± 3.07	1.64 ± 0.15	-0.42 ± 0.06	0.93 ± 0.04	0.92 ± 0.03	0.98 ± 0.01
3T TESS	trochlear latera	4 (3, 7)	36.49 ± 3.50	0.56 ± 0.13	48.58	93.87 ± 18.54	7.83 ± 2.83	0.61 ± 0.09	2895.54 ± 911.	-60.68	2.16 ± 0.41	0.03 ± 0.	3.76 ± 0.	0.47 ± 0.05	0.06 ± 0.01	106.23 ±	19.31 ±	308.97 ± 52.	3.02 ± 0.	8.57 ± 3.25	1.82 ± 0.15	-0.41 ± 0.05	0.92 ± 0.02	0.89 ± 0.02	0.97 ± 0.01

<!--Col Count:26-->T_2 method	Label	Mankin score [a.u.]	Orientation (PLM) [degrees]	Parallelism (PLM) [a.u.]	Mean T_2 [ms]	Autocorrelation	Contrast	Correlation	Cluster prominence	Cluster shade	Dissimilarity	Energy	Entropy	Homogeneity	Maximum probability	Sum of squares	Sum average	Sum variance	Sum entropy	Difference variance	Difference entropy	Information measure of correlation	Information measure of correlation 2	Inverse difference normalized INN	Inverse difference moment normalized
	l				± 3.19				78	± 31.81		00	25			14.24	1.63	14	35						
3T TESS	femoral lateral anterior	5 (3, 9)	35.49 ± 2.92	0.62 ± 0.07	44.90 ± 9.54	77.28 ± 30.32	3.89 ± 2.10	0.77 ± 0.15	4645.33 ± 1647.74	64.09 ± 123.76	1.18 ± 0.43	0.04 ± 0.01	3.59 ± 0.15	0.64 ± 0.07	0.10 ± 0.02	64.96 ± 22.49	14.40 ± 2.87	183.51 ± 70.78	2.85 ± 0.20	3.90 ± 2.49	1.37 ± 0.22	-0.51 ± 0.05	0.95 ± 0.02	0.93 ± 0.03	0.98 ± 0.01
3T TESS	femoral lateral central	5 (4, 7)	34.02 ± 1.99	0.60 ± 0.09	51.26 ± 7.17	96.23 ± 22.16	6.61 ± 2.09	0.63 ± 0.10	1988.78 ± 541.96	-62.31 ± 37.90	1.84 ± 0.33	0.03 ± 0.00	3.66 ± 0.19	0.50 ± 0.04	0.07 ± 0.02	110.93 ± 12.55	20.28 ± 1.33	335.59 ± 46.68	2.78 ± 0.17	6.24 ± 2.12	1.68 ± 0.14	-0.38 ± 0.04	0.91 ± 0.02	0.90 ± 0.02	0.97 ± 0.01
3T TESS	femoral lateral posterior	5 (2, 8)	35.36 ± 3.00	0.65 ± 0.07	47.24 ± 3.41	99.52 ± 18.92	6.37 ± 3.16	0.73 ± 0.14	2861.19 ± 915.22	-19.56 ± 59.35	1.75 ± 0.65	0.03 ± 0.01	3.53 ± 0.21	0.51 ± 0.11	0.07 ± 0.02	102.62 ± 18.45	19.08 ± 2.06	303.57 ± 60.38	2.84 ± 0.26	6.18 ± 3.32	1.57 ± 0.24	-0.47 ± 0.05	0.94 ± 0.01	0.90 ± 0.03	0.98 ± 0.01
7T TESS	femoral	5 (3, 12	35.70 ±	0.63 ± 0.0	46.0	99.42 ±	9.00 ± 4.5	0.57 ±	2829.67 ±	-79.6	2.15 ±	0.04 ±	3.55 ±	0.47 ±	0.09 ± 0.0	120.99	20.96	371.80 ±	2.75 ±	8.90 ± 4.0	1.76 ± 0.1	-0.44 ±	0.93 ±	0.89 ±	0.97 ±

<!--Col Count:26-->T_2 method	Label	Mankin score [a.u.]	Orientation (PLM) [degrees]	Parallelism (PLM) [a.u.]	Mean T_2 [ms]	Autocorrelation	Contrast	Correlation	Cluster prominence	Cluster shade	Dissimilarity	Energy	Entropy	Homogeneity	Maximum probability	Sum of squares	Sum average	Sum variance	Sum entropy	Difference variance	Difference entropy	Information measure of correlation	Information measure of correlation 2	Inverse difference normalized INN	Inverse difference moment normalized
	medial posterior	)	2.57	6	7 ± 8.50	31.91	7	0.11	2145.38	7 ± 83.18	0.52	0.01	0.27	0.06	2	± 19.24	± 2.26	71.97	0.15	6	9	0.05	0.02	0.03	0.01
7T TESS	femoral medial central	6 (2, 8)	33.86 ± 4.53	0.60 ± 0.11	51.88 ± 8.46	92.31 ± 34.52	9.17 ± 3.33	0.43 ± 0.11	1556.18 ± 550.60	-31.56 ± 35.74	2.21 ± 0.39	0.04 ± 0.01	3.55 ± 0.27	0.47 ± 0.05	0.08 ± 0.02	103.89 ± 26.57	19.07 ± 2.96	303.17 ± 89.03	2.67 ± 0.16	9.27 ± 2.81	1.83 ± 0.13	-0.40 ± 0.04	0.91 ± 0.03	0.88 ± 0.02	0.96 ± 0.01
7T TESS	femoral medial anterior	4 (2, 8)	33.73 ± 4.99	0.62 ± 0.12	36.78 ± 7.63	81.85 ± 19.55	4.89 ± 2.85	0.87 ± 0.04	4265.07 ± 1518.82	69.47 ± 75.35	0.90 ± 0.23	0.07 ± 0.02	3.27 ± 0.31	0.72 ± 0.05	0.15 ± 0.05	72.70 ± 16.22	15.41 ± 2.13	212.75 ± 54.38	2.76 ± 0.17	2.99 ± 1.26	1.19 ± 0.16	-0.58 ± 0.03	0.96 ± 0.01	0.93 ± 0.04	0.99 ± 0.00
7T TESS	trochlear medial	5 (4, 9)	34.95 ± 2.91	0.59 ± 0.09	39.53 ± 7.53	79.84 ± 21.78	9.10 ± 1.79	0.49 ± 0.15	2059.11 ± 1133.17	-5.68 ± 79.96	1.95 ± 0.26	0.04 ± 0.01	3.62 ± 0.24	0.51 ± 0.03	0.09 ± 0.02	90.41 ± 19.81	17.90 ± 2.47	267.67 ± 67.67	2.66 ± 0.12	8.10 ± 1.99	1.72 ± 0.11	-0.38 ± 0.05	0.89 ± 0.03	0.91 ± 0.04	0.97 ± 0.01
7T TES	trochl	5 (3,	34.38 ±	0.55 ±	46.	75.65 ±	6.06 ±	0.78 ±	3162.51	13.4	1.46 ±	0.03	3.61	0.56 ±	0.08 ±	79.7	16.1	218.1	2.90	4.46 ±	1.52 ±	-0.46	0.94 ±	0.92 ±	0.98 ±

<!-- Col Count:26 -->T_2 method	Label	Mankin score [a.u.]	Orientation (PLM) [degrees]	Parallelism (PLM) [a.u.]	Mean T_2 [ms]	Autocorrelation	Contrast	Correlation	Cluster prominence	Cluster shade	Dissimilarity	Energy	Entropy	Homogeneity	Maximum probability	Sum of squares	Sum average	Sum variance	Sum entropy	Difference variance	Difference entropy	Information measure of correlation	Information measure of correlation 2	Inverse difference normalized INN	Inverse difference moment normalized
S	ear central	9)	2.63	0.15	90 ± 7.93	26.52	3.51	0.09	± 979.05	3 ± 78.45	0.25	± 0.00	± 0.21	0.04	0.01	1 ± 17.19	4 ± 2.21	4 ± 57.72	± 0.10	1.51	0.13	± 0.04	0.02	0.03	0.01
7T TESS	trochlear lateral	4.5 (3, 7)	36.21 ± 3.42	0.55 ± 0.13	53.06 ± 4.13	92.06 ± 28.51	7.42 ± 1.75	0.61 ± 0.13	2709.76 ± 1147.70	-63.14 ± 48.73	1.97 ± 0.23	0.03 ± 0.01	3.60 ± 0.28	0.51 ± 0.03	0.08 ± 0.02	108.96 ± 18.02	19.60 ± 1.97	322.72 ± 69.13	2.80 ± 0.18	7.82 ± 1.63	1.77 ± 0.08	-0.43 ± 0.04	0.92 ± 0.02	0.89 ± 0.01	0.97 ± 0.01
7T TESS	femoral lateral anterior	5 (3, 9)	35.91 ± 3.06	0.62 ± 0.07	35.94 ± 10.59	86.60 ± 28.01	4.71 ± 2.62	0.77 ± 0.14	3322.99 ± 1896.89	63.31 ± 88.22	1.19 ± 0.40	0.05 ± 0.01	3.30 ± 0.22	0.64 ± 0.09	0.13 ± 0.04	75.46 ± 18.40	16.08 ± 2.25	221.84 ± 61.96	2.74 ± 0.08	4.01 ± 2.02	1.35 ± 0.19	-0.53 ± 0.08	0.95 ± 0.02	0.93 ± 0.02	0.98 ± 0.01
7T TESS	femoral lateral central	5 (4, 7)	34.55 ± 2.52	0.59 ± 0.09	47.00 ± 6.42	88.14 ± 26.25	7.29 ± 2.32	0.52 ± 0.09	1450.47 ± 722.76	-43.63 ± 24.11	1.92 ± 0.40	0.04 ± 0.01	3.50 ± 0.15	0.50 ± 0.05	0.09 ± 0.02	113.01 ± 22.14	20.15 ± 2.41	338.60 ± 71.57	2.67 ± 0.11	7.29 ± 2.90	1.72 ± 0.15	-0.39 ± 0.05	0.90 ± 0.03	0.90 ± 0.02	0.97 ± 0.01
7T	fe	4.	35.1	0.6	4	106.9	6.0	0.71	319	-	1.62	0.	3.	0.56	0.1	11	20	34	2.	6.2	1.5	-	0.94	0.92	0.98

<!--ColCount:26-->T_2 method	Label	Mankin score [a.u.]	Orientation (PLM) [degrees]	Parallelism (PLM) [a.u.]	Mean T_2 [ms]	Autocorrelation	Contrast	Correlation	Cluster prominence	Cluster shade	Dissimilarity	Energy	Entropy	Homogeneity	Maximum probability	Sum of squares	Sum average	Sum variance	Sum entropy	Difference variance	Difference entropy	Information measure of correlation	Information measure of correlation 2	Inverse difference normalized INN	Inverse difference moment normalized
TESS	moral lateral posterior	5 (2, 8)	9 ± 2.88	3 ± 0.08	2.46 ± 7.47	7 ± 27.58	4 ± 2.66	± 0.10	3.92 ± 1487.12	67.51 ± 49.99	± 0.41	04 ± 0.01	46 ± 0.16	± 0.08	0 ± 0.02	4.99 ± 18.06	.27 ± 1.98	8.97 ± 63.80	80 ± 0.12	4 ± 2.54	7 ± 0.19	0.49 ± 0.08	± 0.03	± 0.02	± 0.01

7T (TESS) N = 10.

3T (TESS) N = 9.

3T (MESE) N = 8.
